# Early Signs of Pathological Cognitive Aging in Mice Lacking High-Affinity Nicotinic Receptors

**DOI:** 10.3389/fnagi.2016.00091

**Published:** 2016-04-27

**Authors:** Eleni Konsolaki, Panagiotis Tsakanikas, Alexia V. Polissidis, Antonios Stamatakis, Irini Skaliora

**Affiliations:** ^1^Neurophysiology Laboratory, Center for Basic Research, Biomedical Research Foundation of the Academy of AthensAthens, Greece; ^2^Psychology Department, DEREE-The American College of GreeceAthens, Greece; ^3^Biology-Biochemistry Lab, School of Health Sciences, University of AthensAthens, Greece

**Keywords:** cholinergic, β2-nAChR, habituation, cognition, animal model, behavioral phenotype, premature aging, exploration

## Abstract

In order to address pathological cognitive decline effectively, it is critical to adopt early preventive measures in individuals considered at risk. It is therefore essential to develop approaches that identify such individuals before the onset of irreversible dementia. A deficient cholinergic system has been consistently implicated as one of the main factors associated with a heightened vulnerability to the aging process. In the present study we used mice lacking high affinity nicotinic receptors (β2-/-), which have been proposed as an animal model of accelerated/premature cognitive aging. Our aim was to identify behavioral signs that could serve as indicators or predictors of impending cognitive decline. We used test batteries in order to assess cognitive functions and additional tasks to investigate spontaneous behaviors, such as species-specific activities and exploration/locomotion in a novel environment. Our data confirm the hypothesis that β2-/- animals exhibit age-related cognitive impairments in spatial learning. In addition, they document age-related deficits in other areas, such as recognition memory, burrowing and nesting building, thereby extending the validity of this animal model for the study of pathological aging. Finally, our data reveal deficits in spontaneous behavior and habituation processes that precede the onset of cognitive decline and could therefore be useful as a non-invasive behavioral screen for identifying animals at risk. To our knowledge, this is the first study to perform an extensive behavioral assessment of an animal model of premature cognitive aging, and our results suggest that β2-nAChR dependent cognitive deterioration progressively evolves from initial subtle behavioral changes to global dementia due to the combined effect of the neuropathology and aging.

## Introduction

Age-related cognitive impairment and dementia are increasingly problematic both for individuals and for societies (Fratiglioni et al., 2000), emphasizing the need for understanding the mechanisms that distinguish normal from pathological aging and designing appropriate therapeutic interventions. In principle, there are two ways to address the issue of age-related cognitive decline: the first is to develop intervention strategies for the reversal of already established symptoms (Broadstock et al., 2014; Gant et al., 2015; Wootla et al., 2015). Although this is clearly an important approach, evidence so far suggests that it is usually not very effective, as the neurobiological substrate may be too compromised to allow amelioration of the symptoms (Rafii and Aisen, 2009). The alternative approach is to develop strategies to prevent, or delay age-associated cognitive decline before it becomes settled, thereby increasing the chances to produce longer lasting effects (Galeano et al., 2014; Wisniewski and Drummond, 2015). Such strategies may include not only pharmacological treatments but also changes in diet and lifestyle to modify brain function before the onset of irreversible dementia (Mangialasche et al., 2012). In order to pursue the latter approach it is essential to use animal models that exhibit age-associated cognitive impairments and examine them earlier in life in order to identify risk factors for pathological cognitive aging.

Given the recognized role of the nicotinic system in cognition and age-related deficits (Sarter and Bruno, 2004; Maskos et al., 2005; Lombardo and Maskos, 2015), one such animal model is the β2 knockout mouse (β2-/-), in which the β2-containing high-affinity nicotinic receptors (β2-nAChRs) have been genetically deleted. These mice have been suggested as model of accelerated/premature cognitive aging on the basis of deficits in spatial learning and concomitant alterations in the structure of certain brain regions that are manifested only in aged animals (Zoli et al., 1999). Hence, this animal model could be exploited to search for early indications that could presage sub-clinical brain dysfunction and promote accelerated cognitive decline. Indeed, anatomical studies have revealed changes in pyramidal cell morphology already present in adulthood, that become particularly accentuated with age, supporting the view that pathological aging could have its roots early in life (Ballesteros-Yáñez et al., 2010; Konsolaki and Skaliora, 2015b). Nevertheless, such morphological indications cannot be used to screen for animals at risk of developing dementia, as they require postmortem evaluation.

In the present study we undertook an extensive assessment of adult and old β2-/- mice, in order to identify behavioral differences that could serve as indicators or predictors of impending cognitive decline. We used a broad combination of tasks, to investigate not only standard cognitive functions but also spontaneous behaviors, such as locomotion and habituation to a novel environment; and species-specific activities such as hoarding, burrowing, marble burying, and nest construction (Deacon et al., 2003, 2008, 2012). In addition, we employed mildly/minimally enriched housing conditions to avoid the confounding factor of the severely deprived environment usually associated with standard rearing protocols (Mo et al., 2015).

Our results confirm and extend the hypothesis that β2-/- animals show age-related cognitive impairments, which are manifested not only in spatial learning, but also in recognition memory tasks; and further reveal deficits in spontaneous behavior and habituation processes earlier in life. To our knowledge this is the first study to perform an extensive behavioral examination of an animal model of premature cognitive aging and our data suggest that the β2-nAChR dependent cognitive deterioration progressively evolves from initial subtle behavioral changes to global dementia due to the combined effect of the neuropathology and aging.

## Materials and Methods

### Ethics Statement

The present study was approved by the Regional Veterinary Service, in accordance to the National legal framework for the protection of animals used for scientific purposes (reference number 2834/08-05-2013).

### Mice

Adult (4–6 months) and old (22–24 months) male wild type (WT) and β2-/- C57Bl/6J mice were used in the present study and were bred in the animal facility of the Center for Experimental Surgery of the Biomedical Research Foundation of the Academy of Athens from animals that were obtained from the University of California at Davis (generous gift of Professor Leo Chalupa). Animals were genotyped using tail snips to confirm the absence of the β2 subunit using the following primers: (1) 5′-CGGAGCATTTGACTCTGAGCAGTGGGGTCGC-3′, (2) 5′-CTCGCTGACACAAGGGCTGCGGAC-3′, (3) 5′-CTTGGGTGGAGAGGCTATTC-3′, (4) 5′-AGGTGAGATGACAGGAGATC-3′. The WT and mutant products are 360 and 280 bp, respectively. All animals were housed in groups (4–6 per cage) in nesting-enriched cages with corncob bedding under a regular 12-h light/dark cycle at controlled room temperature (22 ± 2°C) and humidity (55 ± 10%). The facility is registered as a breeding and experimental facility according to the Presidential Decree of the Greek Democracy 160/91, which harmonizes the Greek national legislation with the European Council Directive 86/609/EEC on the protection of animals used for experimental and other scientific purposes. All animals in the animal facility were screened for viral and bacterial infections and parasites every 3 months using a health-monitoring program in accordance with the recommendations of the Federation of European Laboratory Animal Science Associations (Nicklas et al., 2002). Before, all behavioral experiments (except the assessment of species-specific behaviors) animals were handled for 7 days for 1–2 min/day in order to become familiarized with the experimenter and minimize stress during the tasks.

Behavioral assessment was performed on three groups of experimental animals as follows: (i) the first group was tested in the open field and the elevated plus maze with a 24 h interval between the two (WT adult *n* = 10; WT old *n* = 6; β2-/- adult *n* = 10; β2-/- old *n* = 12); (ii) the second group was tested in species-specific behaviors (burrowing, hoarding, and nest construction), followed by novel object recognition and Morris water maze (MWM) tasks, separated by a minimum 5-days interval between tests (WT adult *n* = 8; WT old *n* = 11; β2-/- adult *n* = 11; β2-/- old *n* = 11); (iii) the third group was tested for marble burying (WT adult *n* = 12; WT old *n* = 7; β2-/- adult *n* = 12; β2-/- old *n* = 9).

### Behavioral Assessment

#### Open Field

One hour prior to the open field test, the mice were transferred to the experimental room for acclimatization. They were placed in a plexiglass box (40 cm × 30 cm × 20 cm), between 10 am and 3 pm and observed for a 1-h period, under normal lighting conditions (150 lux). The animals’ locomotion (horizontal and vertical) was recorded by two cameras connected to an electronic monitoring system (Ethovision XT8, Noldus Information Technology, Utrecht, The Netherlands). The distance traveled (in centimeters) and the number of rears was measured. Two-way ANOVA (60 min) and three-way repeated-measures ANOVA (15, 30 min) were performed, in order to assess the animals’ overall mobility and habituation to a novel environment, respectively.

In order to further investigate the spatiotemporal organization of locomotor behavior in a novel environment, two different types of displacements were estimated: fast, large movements (navigation) and slow, local movements (exploration) according to published protocols (Granon et al., 2003; Maskos et al., 2005; Avale et al., 2008). The instantaneous velocity (cm/sec) was exported for each video file of each animal, and an excel file was created that included the velocity time-series for every animal within the same age-and-genotype group: WT adult, WT old, β2-/- adult, and β2-/- old mice. Locomotor activity was scaled based on velocity and classified into three different categories: exploration, navigation, intermediate (Supplementary text and **Figure S1**). Three-way repeated-measures ANOVA (15, 30 min) and two-way ANOVA (60 min) were performed, in order to assess navigation and exploration related to habituation and locomotion in the novel environment, respectively.

#### Elevated Plus Maze

The elevated plus maze, made of PVC with a matte white surface, consisted of four arms, 6 cm wide and 28 cm long, elevated 40 cm above the floor (Panlab, Harvard Apparatus). Two opposing arms were enclosed by 15-cm-high walls, whereas the other two were open. Each mouse was placed in the central platform facing an open arm (File et al., 2004) and was allowed to roam freely about the maze for 5 min. The mouse behavior was recorded by a video camera mounted directly above the maze. We measured time in open arms/time in open and closed arms, as an index of anxiety. An arm entry was scored if an animal entered an arm with all four paws. Statistical evaluation was performed with two-way ANOVA.

#### Assessment of Species-Specific Behaviors

Four different tasks were examined in order to evaluate the animals’ spontaneous behavior. Animals experienced isolation from cagemates at the same time, as cagemates were subjected simultaneously to task sessions. In addition, pseudorandomized placement into testing cages was performed.

##### Burrowing

At least 2 h before the start of the dark period, mice (not food deprived) were placed in individual polysulfone cages 42.5 cm × 26.6 cm × 18.5 cm (Techniplast, Buguggiate, Italy) with corncob bedding, containing a tube (white plastic, 22 cm long, 6.5 cm diameter) filled with 250 g of normal diet food pellets. The lower end was sealed, resting on the cage floor. The open end was supported 3.5 cm above the floor to prevent the contents from being non-purposefully displaced. A cardboard tube (20 cm long, 7 cm diameter) with the lower end closed was also placed in the test cage, to provide an alternative hiding place for mice that merely sought refuge in the burrowing tube (Deacon, 2006c). Water was provided in the cages.

The weight and the number of pellets remaining in the tube were measured in the morning. The food consumed (between 2 and 4 g on average) is assumed to be identical to all mice. Two-way ANOVA was performed to evaluate the effect of age and genotype on burrowing performance.

##### Hoarding

On the day of the assessment mice were individually placed in polysulfone cages 36.5 cm × 20.7 cm × 14 cm (Techniplast, Buguggiate, Italy) with a mouse igloo (BioServ, The Netherlands) and corncob bedding. Each cage was attached to a wire mesh tube (60 cm), at the far end of which were placed 100 g of normal diet food pellets (each weighing around 2 g) for the first dark phase, and 180 g of food pellets for the second dark phase. Water and food (3 g) was provided inside the cages. In the morning of the first test day mice were placed in the cages with the hoarding tube entrances blocked, in order to habituate. The tubes were opened by late afternoon, just before the start of the dark phase (Deacon, 2006a). The next morning the pellets inside the cage, as well as those remaining in each tube, were weighed and counted. The hoarding test was repeated on the following day. Data were analyzed with non-parametric statistical test (Mann–Whitney) as the distribution of hoarding performance values deviated from normality according to the Shapiro–Wilk test.

##### Nest construction

Mice were placed individually in polysulfone cages (42.5 cm × 26.6 cm × 18.5 cm) with corncob bedding about 1 h before the dark phase. Seven pieces of pressed cotton (5 cm × 3.5 cm squares; Pur-Zellin Hartman, Germany) were placed in the center of each cage and nesting behavior was assessed the next morning. The nests were scored by two independent observers blind to the group identity according to the following scale: 0 = undisturbed; 1 = disturbed; 2 = flat nest; 3 = cup-shaped nest; 4 = incomplete dome; 5 = complete dome (Hess et al., 2008). Data were analyzed with the non-parametric Mann–Whitney test, as the distribution of nest scores deviated from normality, according to Shapiro–Wilk test.

##### Marble burying

One hour prior to the test, the mice were transferred to the experimental room for acclimatization. A plexiglass box (40 cm × 40 cm × 35 cm) whose sides were covered with white cardboard was filled with 4 cm of corncob bedding (pressed down to form a level surface) on top of which 49 glass marbles (15 mm diameter) were placed in a 7 × 7 array. Bedding from the animals’ cages was added to the box. Mice were placed in the box and their behavior was recorded for 30 min. At the end of this period, the number of marbles buried (to at least 2/3 of their depth) was counted (Thomas et al., 2009). Two-way ANOVA was performed to evaluate the effect of age and genotype on marble burying performance.

#### Novel Object Recognition Task

The novel object recognition task (NORT) was conducted in a 40 cm × 40 cm × 35 cm plexiglass box (whose sides were covered with white cardboard) filled with corncob bedding up to 1 cm. Bedding from the mice cages was added to the box. A video camera was mounted in the ceiling, and the mouse’s behavior was recorded for off-line analysis.

The task was divided into three distinct phases over three consecutive days: habituation, sample, and discrimination phases (Pires et al., 2009). Each trial lasted 10 min and trials were separated by a 10 min interval. The habituation phase (day 1) consisted in exploration of the box (two trials). The sample phase consisted in exploration of two identical objects, and took place once after 24 h (day 2) and once after 48 h (day 3): in this phase, two identical objects (either two 7 cm × 10 cm × 12 cm building blocks or two 8 cm × 10 cm × 12 cm milk cans) were placed at a distance of 12 cm from the box corners. Exploration of the object was defined as facing and sniffing the object at a distance of less than 1 cm and actively exploring it. The discrimination phase occurred after the second sample phase on day 3, where one of the familiar objects was replaced by a novel object. Object locations were counterbalanced and both cage and objects were cleaned after trials to eliminate odor cues. The discrimination index (time exploring the novel object - time exploring the familiar object)/(time exploring the novel object + time exploring the familiar object) was measured and two-way ANOVA was performed to evaluate the differences in the time exploring the familiar vs. the non-familiar object among the four experimental groups.

#### Morris Water Maze Task

The MWM task (Morris, 1984) took place in a circular pool (140 cm in diameter, 60 cm height) filled with tap water (24 ± 1°C). The water was made opaque with the addition of 100 ml of non-toxic white liquid tempera paint (Reeves, England) to ensure proper camouflage of the escape platform. The escape platform was constructed from a plexiglass cylinder (14 cm in diameter, 40 cm height) and the water level was 1 cm above the platform, rendering it invisible. For the cued acquisition a bright and colorful flag was placed on the platform. The pool was located in a room with 2D high-contrast posters around the walls, which served as extra-maze visual cues. We used static shapes with high contrast avoiding vertical lines in order to eliminate possible effects on the performance of β2-/- mice (Wang et al., 2009). The animals’ performance was recorded by an electronic monitoring system connected to a camera.

The water maze protocol was divided into three phases: cued acquisition, acquisition and probe trial, reversal learning and reverse probe trial (Vorhees and Williams, 2006). During the cued acquisition, we assessed the ability of the mice to learn to swim to and climb onto the visible, flagged platform in the absence of extra maze cues. The platform was moved to a different position for each 1 min trial. Cued learning consisted of two training sessions over two consecutive days; each session included four trials (max duration 1 min) separated by a 15 min inter-trial interval. During the acquisition phase (four trials/day for 6 days), mice were trained to swim to a hidden platform that was located in the southwest quadrant. Each mouse was released from a randomly assigned start location (East, North, South, or West) facing the wall. If the mouse did not find the platform within 60 s, it was guided to it and allowed to stay on the platform for 20 s. The inter-trial interval was 15 min. On day 9, mice were tested for spatial memory in a 60 s probe trial with no platform present.

In addition to the conventional latency analysis we also examined the trajectories the mice followed during the acquisition phase in order to assess their learning strategies. Previous studies have classified search strategies in seven distinct categories based on the spatial characteristics of the trajectories: thigmotaxis, random search, scanning, chaining directed search, focal search, and directed swimming (Garthe et al., 2009; Ruediger et al., 2012). Here, we performed the same analysis using a programmable software (BIOBSERVE), and subsequently combined these strategies into three groups: escape strategies (thigmotaxis and random search); local strategies (scanning, chaining) and global strategies (focal search, directed swimming). The percent of the trajectory preference in each trial was calculated and repeated measures ANOVA was performed for statistical evaluation of the data.

During reversal training (four trials/day for 5 days) the platform was moved to the opposite quadrant (Northeast) while the protocol was identical to the one described for the regular training. On day 15, mice were tested for spatial memory in a 60-s reverse probe trial with no platform present. The latency differences in finding the hidden platform during the acquisition and reversal training phases were evaluated with three-way-repeated-measures ANOVA. The probe trial performance was evaluated as number of crossings over the platform position. Since these data deviate from normality, they were first transformed using the Box–Cox transformation (Box and Cox, 1964) to become normally distributed and with equal variance, and ANOVA was applied on the transformed data.

## Results

### β2-/- Mice Exhibit Deficits in Spatial Learning and Memory

We first examined the animals’ performance in the MWM task to evaluate/confirm the accelerated aging phenotype of β2-/- mice. We used a 140 cm diameter pool and recorded the animals’ trajectories through an electronic monitoring system connected to a camera. Mice were trained in the cued phase (with a visible platform and no extra-maze cues) for 2 days to become familiarized with the task and exclude any visual deficits. All mice exhibited similar latencies to escape onto the visible platform [day × genotype × age: *F*(1,36) = 1.679; *p* = 0.203, day × age: *F*(1,36) = 0.248; *p* = 0.622, day × genotype: *F*(1,36) = 2.109; *p* = 0.155; day effect: *F*(1,36) = 72.528, *p* < 0.001] indicating they all had normal visual and motor function.

**FIGURE 1:**
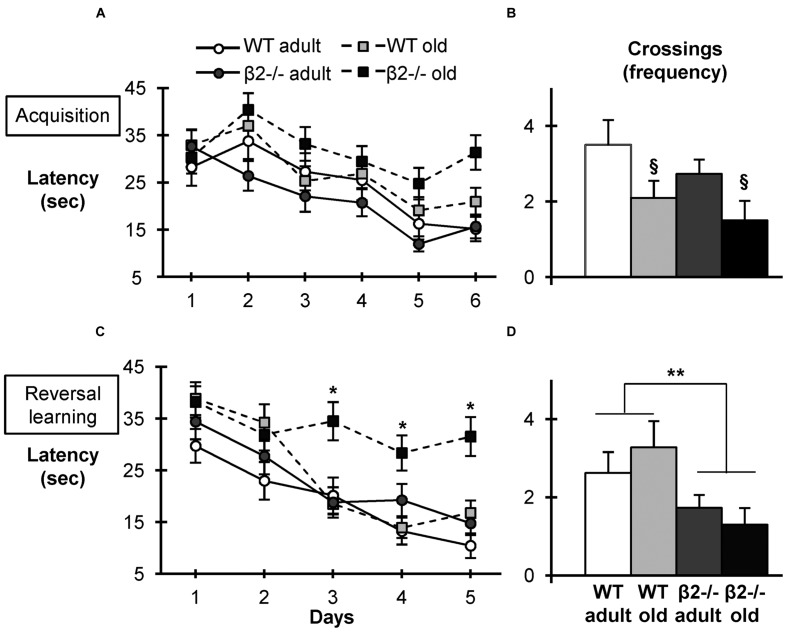
**Acquisition, Reversal Learning, and probe tests in the Morris Water Maze Task.** **(A)** Latency to find the hidden platform during the 6-days acquisition phase. **(B)** Crossings over the initial position of the platform during the probe test. **(C)** Latency to find the hidden platform during the reverse learning phase (opposite quadrant). **(D)** Crossings over the new position of the platform during the reversal probe test. Data are represented as mean ± SEM; symbols represent significant age (^x^*p* < 0.005) and genotype (^∗^*p* < 0.005; ^∗∗^*p* < 0.001) differences.

During the 6-days acquisition phase, all groups of mice exhibited similar learning curves in finding the hidden platform using extra-maze cues [latency: day × genotype × age: *F*(5,180) = 0.647, *p* = 0.664, day × age: *F*(5,180) = 0.730, *p* = 0.602, day × genotype: *F*(5,180) = 0.46, *p* = 0.802; day effect: *F*(5,180) = 11.088, *p* < 0.001; distance: day × genotype × age: *F*(5,180) = 0.682, *p* = 0.638, day × age: *F*(5,180) = 0.884, *p* = 0.493, day × genotype: *F*(5,180) = 0.802, *p* = 0.549; day effect: *F*(5,180) = 11.966, *p* < 0.001]. Consistent with an accelerated aging phenotype (Picciotto et al., 1995; Zoli et al., 1999), there was a tendency for longer latencies in aged β2-/- mice, but the difference did not reach significance levels (**Figure 1A**). Similarly, the probe test performed after the completion of the acquisition phase revealed the expected effect of age on crossings frequency [*F*(1,36) = 7.080; *p* = 0.012], but no effect of genotype [*F*(1,36) = 1.678; *p* = 0.203; **Figure 1B**], indicating that β2-/- mice perform as well WT age-matched animals.

These findings appear to contradict previous data on age-related spatial learning impairments in β2-/- mice, although a direct comparison is precluded by the differences in experimental conditions, including size of the pool, duration of the acquisition phase and statistical analysis (Zoli et al., 1999). We hypothesized the discrepancy could be due to the mildly enriched housing conditions (Jankowsky et al., 2005). So we proceeded to test mice in the more challenging reverse water maze task, which also assesses cognitive flexibility and requires the development of new spatial strategy (Vorhees and Williams, 2006, 2014). The hidden platform was moved to the opposite quadrant and mice were trained to find the new target using the same spatial cues. Indeed, in this task aged β2-/- mice displayed significantly reduced performance in the acquisition phase compared to the other three groups [day × genotype × age: *F*(4,144) = 3.576; *p* = 0.008, *post hoc* days 3, 4, and 5; *p* < 0.005; **Figure 1C**]. Notably, the probe test revealed the memory deficit in this task was already present in adult mutant animals [genotype: *F*(1,36) = 8.203; *p* = 0.007; age: *F*(1,36) = 0.002; *p* = 0.967; **Figure 1D**]. Taken together these results indicate that although aged β2-/- mice display normal spatial learning, they show marked deficits in reversal learning (**Figure 1C**), consistent with an accelerated aging phenotype.

**FIGURE 2:**
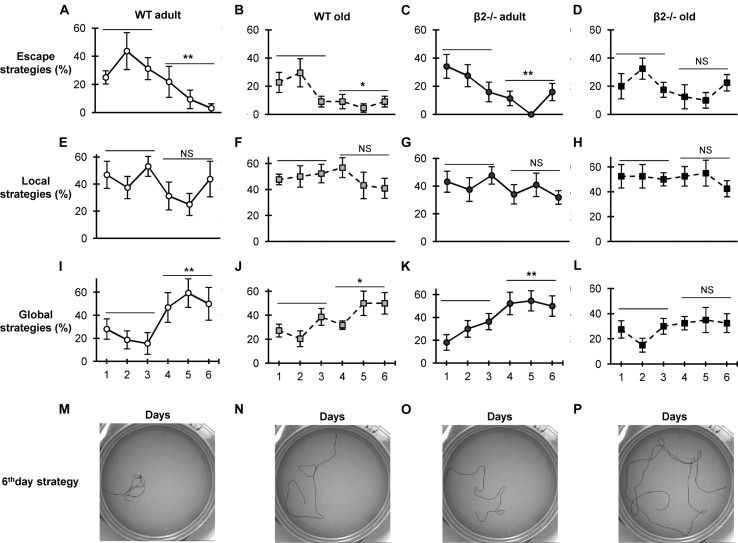
**Strategy preference during the acquisition phase in the Morris Water Maze Task.** Data are presented as percentages of the different strategies for each day of the acquisition phase. **(A–D)** Escape strategies (thigmotaxis and random search); **(E–H)** local strategies (scanning, chaining). **(I–L)** Global strategies (focal search, directed swimming); **(M–P)** examples of the representative trajectories for each group on the 6th day. Data are represented as mean ± SEM; symbols represent significant (^∗^*p* < 0.005; ^∗∗^*p* < 0.001) and non-significant (NS) difference between days.

Given the mismatch between the aged β2-/- animals’ performance for the first and second location, we investigated the learning strategies employed by the animals to locate the platform. Previous studies have established that separate brain networks implement reinforced learning through the recruitment of distinct behavioral search strategies as mice learn the MWM task, and have classified them into seven categories based on the spatial characteristics of the trajectories (Garthe et al., 2009; Ruediger et al., 2012). Indeed, when we analyzed the trajectories we found WT as well as adult β2-/- animals showed a clear progression from escape strategies (thigmotaxis and random search) to global strategies (focal search and directed swimming; **Figure 2**). In contrast, old β2-/- mice were the only group that was not able to shift to global strategies after the first 3 days [escape strategies: WT adult *F*(5,35) = 4.412, *p* = 0.003, WT old *F*(5,50) = 3.508, *p* = 0.042, β2-/- adult *F*(5,45) = 3.527, *p* = 0.009, β2-/- old *F*(5,45) = 1,450, *p* = 0.225; global strategies: WT adult *F*(5,35) = 3.948, *p* = 0.006, WT old *F*(5,50) = 2.781, *p* = 0.027, β2-/- adult *F*(5,45) = 4.203, *p* = 0.003, β2-/- old *F*(5,45) = 1.333, *p* = 0.268].

Taken together, these results indicate that although aged β2-/- mice display similar spatial acquisition and reference memory, they have altered learning strategies and show marked deficits in reversal learning, consistent with a pathological aging phenotype. The data further show that the deficit in the reverse reference memory (probe test) is already evident in adulthood.

### Aged β2-/- Mice Exhibit Deficits in Recognition Memory

**FIGURE 3:**
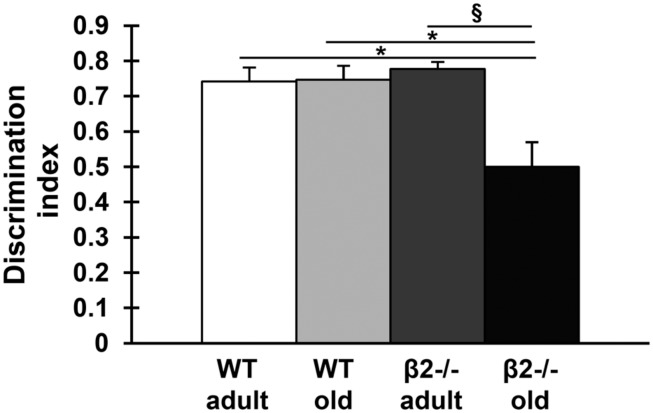
**Performance of the four experimental groups in the novel object recognition task.** Data (mean ± SEM) are presented as discrimination index values (time exploring novel object-time exploring familiar object/total time exploring objects). Symbols represent significant age (^x^*p* < 0.005) and genotype (^∗^*p* < 0.005) differences in exploring the familiar vs. the non-familiar object.

Having confirmed a deficit in spatial learning consistent with an accelerated/premature/pathological cognitive aging phenotype, we then explored whether other aspects of cognition were also similarly affected. Mice of the four experimental groups were tested on the NORT. All mice demonstrated the same preference to both identical objects as indicated by the discrimination index [age × genotype: *F*(1,33) = 0.310; *p* = 0.581, genotype: *F*(1,33) = 0.270, *p* = 0.607; age: *F*(1,33) = 0.087; *p* = 0.770]. One WT old mouse with outlier values as indicated by boxplots for the discrimination index (not shown) was excluded from the analysis. We found an age effect in total time exploration of the objects as expected [Burke et al., 2010; pretest: age × genotype: *F*(1,33) = 0.186; *p* = 0.669, genotype: *F*(1,33) = 1.946, *p* = 0.172; age: *F*(1,33) = 6.828; *p* = 0.013; test: age × genotype: *F*(1,33) = 1.399; *p* = 0.245, genotype: *F*(1,33) = 0.634, *p* = 0.431; age: *F*(1,33) = 7.005; *p* = 0.012]. We also found that, whereas all WT animals as well as adult β2-/- animals were able to discriminate between novel and familiar object, aged β2-/- mice failed to do so [age × genotype: *F*(1,33) = 4.124; *p* = 0.049; **Figure 3**].

### β2-/- Mice Exhibit Altered Species-Specific Behaviors

**FIGURE 4:**
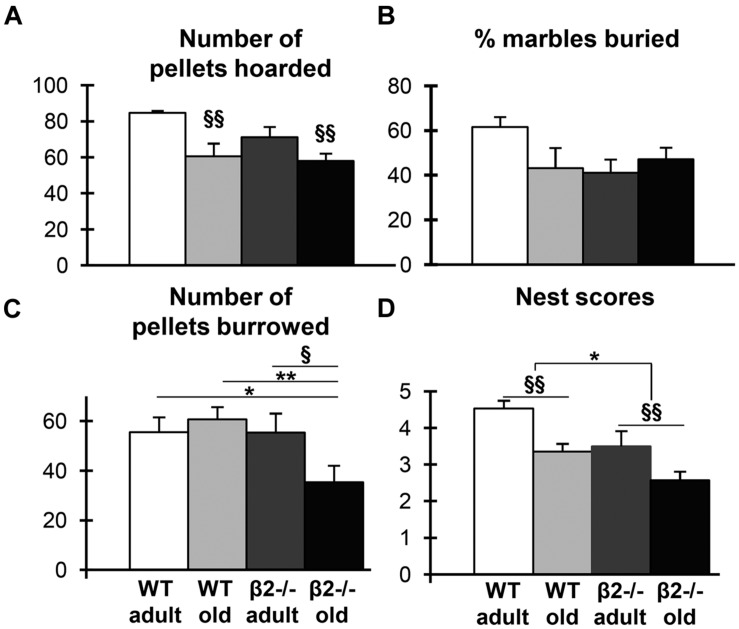
**Species-specific behaviors.** **(A)** Number of pellets hoarded in the second dark phase. **(B)** Percentage of marbles buried in the marble burying test. **(C)** Number of pellets burrowed in the burrowing test. **(D)** Nest scores in the nesting construction assessment. Data are represented as mean ± SEM; symbols represent significant age (^x^*p* < 0.005; ^xx^*p* < 0.001) and genotype (^∗^*p* < 0.005; ^∗∗^*p* < 0.001) differences.

Given that activities of daily living (ADL) are often good predictors of cognitive decline in humans (De Vriendt et al., 2015), we next examined a variety of species-specific spontaneous behaviors in our experimental groups, including hoarding, marble burying, burrowing, and nesting (Deacon, 2006a,b,c,d). We found an age effect on hoarding behavior as old mice of both genotypes showed impairment during both the first (*U* = 120; *p* = 0.045) and the second (*U* = 67.5; *p* < 0.001) dark phase. However, there was no genotype effect, as β2-/- mice were indistinguishable from the control group (**Figure 4A**). Similarly, there was no genotype effect in marble burying behavior, although adult WT animals tended to bury more marbles compared to the other groups [**Figure 4B**; genotype × age: *F*(1,34) = 3.992; *p* = 0.054]. In contrast, burrowing behavior was impaired selectively in old β2-/- mice [**Figure 4C**; genotype × age: *F*(1,36) = 4.171; *p* = 0.049; WT adult vs. β2ko old *p* = 0.04; WT old vs. β2ko old *p* = 0.001; β2ko adult vs. β2ko old *p* = 0.022] consistent with an accelerated aging phenotype. Finally, nesting behavior was significantly affected both by age and by genotype (**Figure 4D**; age: *U* = 96.5, *p* = 0.003; genotype: *U* = 117; *p* = 0.015) so that adult β2-/- animals had nesting scores similar to the aged WT mice. Taken together, these results reveal specific age-dependent deficits in mutant animals in nesting and burrowing, with the former already evident in adulthood. The differences cannot be attributed to anxiety levels, as there was neither age nor genotype effects in the elevated plus maze assessment [age × genotype: *F*(1,32) = 0.629, *p* = 0.434, age: *F*(1,32) = 2.316, *p* = 0.138, genotype: *F*(1,32) = 0.948, *p* = 0.338].

### β2-/- Mice Exhibit Impaired Habituation in a Novel Environment

**FIGURE 5:**
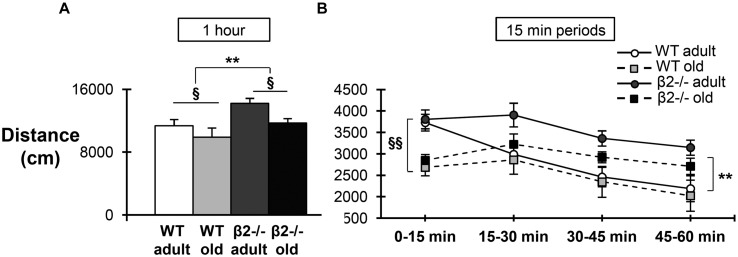
**Locomotion in the open field task.** **(A)** Total distance traveled in centimeters during the 1-h period. **(B)** Distance traveled in centimeters for the four consecutive 15 min periods. Data are represented as mean ± SEM; symbols represent significant age (^x^*p* < 0.005; ^xx^*p* < 0.001) and genotype (^∗∗^*p* < 0.001) effects [triple interaction time × age × genotype: *F*(3,102) = 0.816, *p* = 0.488; time × age: *F*(3,102) = 5.353, *p* = 0.002; time × genotype: *F*(3,102) = 4,578, *p* = 0.005].

Finally, we examined the animals’ performance in the open field task, in order to assess spontaneous behavior in a novel environment. Mice of the four groups were placed in a 40 cm × 30 cm open field arena and their behavior was recorded for 60 min (**Figure 5A**). As expected, there was a significant main effect of age on the total distance traveled [*F*(1,34) = 6.646; *p* = 0.014], reflecting the decreased motility of older animals. In addition, there was also a significant genotype effect in the distance covered during the 1-h period [*F*(1,34) = 9.061; *p* = 0.005], consistent with earlier studies proposing that β2-/- mice have a hyperactive phenotype (Avale et al., 2008; Bourgeois et al., 2012). However, when the distance traveled was calculated separately for consecutive 15 min periods (**Figure 5B**), it became apparent that the initial locomotion does not differ between genotypes [*F*(1,34) = 3.319; *p* = 0.077], but instead that β2-/- mice fail to show the expected habituation over time [*F*(1,34) = 13.289; *p* = 0.001].

**FIGURE 6:**
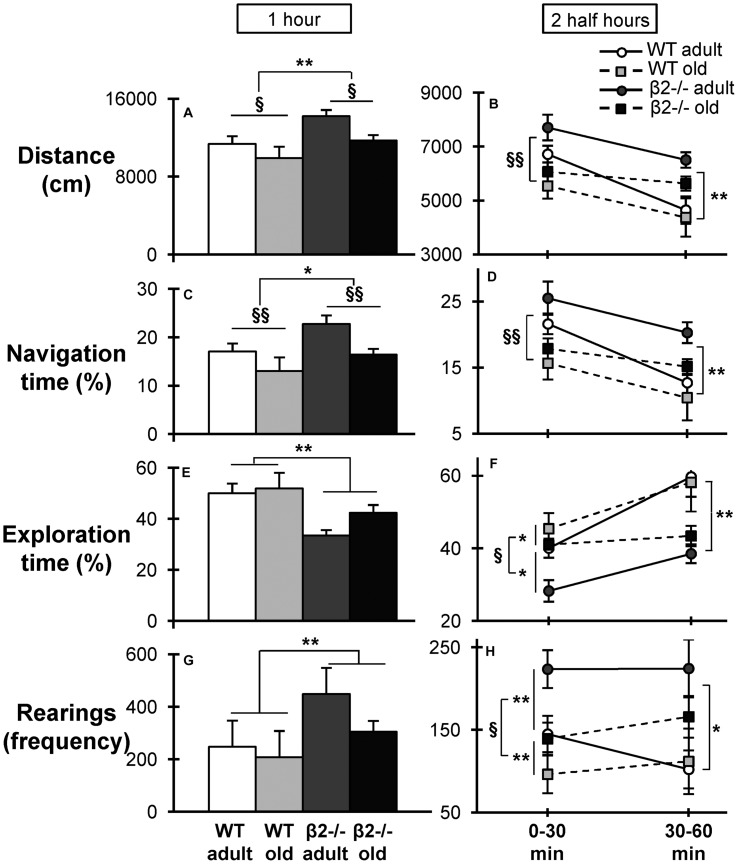
**Distance, navigation, exploration, and rearings in the open field task.** **(A)** Total distance traveled in centimeters during the 1-h period and **(B)** the two half hour periods of the task. Percentage of time spent in navigation during **(C)** the entire 1-h period of the task, and **(D)** the two half hour periods of the task [interaction time × age × genotype: *F*(3,102) = 1.138, *p* = 0.338; time × age: *F*(3,102) = 4.471, *p* = 0.005; time × genotype: *F*(3,102) = 2.6, *p* = 0.05]. Percentage of time spent in exploration during **(E)** the 1-h period, and **(F)** the two half hour periods [interaction time × age × genotype: *F*(3,102) = 0.254, *p* = 0.858; time × age: *F*(3,102) = 4.028, *p* = 0.009; time × genotype: *F*(3,102) = 6.77, *p* < 0.001]. Frequency of the rearings during **(G)** the 1-h period, and **(H)** the two half hour periods. Data are represented as mean ± SEM; symbols represent significant age (^x^*p* < 0.005; ^xx^*p* < 0.001) and genotype (^∗^*p* < 0.005; ^∗∗^*p* < 0.001) differences.

To further explore this inability to habituate in a novel environment we investigated the *patterns* of locomotion by quantifying the time spent in navigation (fast, large movements) vs. exploration (slow, local movements). Previous studies have suggested that navigation behavior reflects the acquisition of general information about the environment while exploratory behavior reflects a more precise investigation of the environment (Granon et al., 2003; Maskos et al., 2005; Avale et al., 2008). Navigation over the entire 1-h period was found to recapitulate the pattern of locomotion, with statistically significant effects of both age [*F*(1,34) = 8.219, *p* = 0.007; **Figure 6A**] and genotype [*F*(1,34) = 6.381, *p* = 0.016; **Figure 6C**]. Interestingly, exploration over the same period manifested only a genotype effect [*F*(1,34) = 12.351, *p* = 0.001; **Figure 6E**], suggesting that β2-/- animals have an altered mobility pattern when interacting with a novel environment.

We next investigated how these patterns change over time by performing three-way repeated measures ANOVA (genotype × age × time) on the different types of movement during consecutive periods of time. For clarity of presentation of the statistical comparisons the data are presented for two 30 min periods. Navigation again showed a similar pattern to total distance covered, with an age effect manifested only during the first half hour [*F*(1,34) = 10.827; *p* = 0.002]; and a genotype effect only during the second half hour [*F*(1,34) = 10.386; *p* = 0.003; **Figures 6B,D**], reflecting the deficient habituation of β2 mutant animals. In contrast, exploration exhibited a significant main effect of genotype [*F*(1,34) = 5.409, *p* = 0.026] already from the first half hour, which persisted in the second half hour [*F*(1,34) = 15.455, *p* < 0.001]; and a main effect of age the first half hour [*F*(1,34) = 6.684, *p* = 0.014; **Figure 6F**]. Importantly, the frequency of rearings, which have been used as a marker of environmental novelty and an index of environmental information acquisition (Thiel et al., 1999; Lever et al., 2006), was also increased in β2-/- mice over the entire 1-h period (**Figure 6G**) [genotype: *F*(1,34) = 8.767, *p* = 0.006] and this difference was apparent both during the first [*F*(1,34) = 7.093, *p* = 0.012] and during the second [*F*(1,34) = 7.524, *p* = 0.010] half hour periods (**Figure 6H**).

Taken together, these results indicate that mice lacking β2-nAChRs have an altered strategy of exploring a novel environment, which is manifested as impaired habituation.

## Discussion

In the present study we examined both cognitive tasks and spontaneous activities in animals lacking high affinity nicotinic receptors in order to identify early behavioral signs that could foretell progressive cognitive deficits. Our data indicate that β2-nAChRs contribute to age-related deficits in learning tasks and species-specific behaviors; and further reveal more subtle changes in spontaneous activities that are present in adulthood, before the cognitive decline sets in. Hence, this work confirms and extends the validity of β2-/- mice as an animal model of pathological cognitive aging and introduces a behavioral screen that may be useful in identifying animals at risk of dementia. Based on such a screen, future studies could explore the effect of early interventions on the progression of cognitive decline with aging.

### β2-/- Mice Exhibit Age-Related Cognitive Deficits

#### Spatial Learning

Although, the cholinergic system is heavily involved in spatial learning (Deiana et al., 2011; Okada et al., 2015), loss of high-affinity nicotine receptors *per se* is not sufficient to cause learning deficits, as evidenced by the normal performance of young and adult β2-/- mice on the MWM task (Picciotto et al., 1995; Zoli et al., 1999). Instead, it seems that the chronic absence of high-affinity nAChRs gradually leads to alterations in brain circuits (Harrist et al., 2004) that ultimately contribute to the decreased performance with aging. The first goal of this study was to evaluate the age-related decline in spatial learning and memory in these animals. Although, earlier investigations had reported significant impairments in aged β2-/- mice in the hidden platform task as compared with their control siblings (Zoli et al., 1999), our data did not replicate this finding. The discrepancy could be attributed to differences in the experimental protocol, such as size of pool, duration of training, or different statistical analysis (i.e., two-way vs. three-way repeated measures ANOVA). Alternatively, it is possible that the use of minimally enriched housing conditions employed in our study curtailed the effects of aging on spatial learning and memory, as has been shown for the full enrichment protocol (Frick et al., 2003; Bennett et al., 2006; Harburger et al., 2007). Consistent with this interpretation, we did not observe an age-related deficit in the water maze performance of our WT animals, as would have been expected based on previous studies (Bellush et al., 1996; Magnusson et al., 2003; van Praag et al., 2005; Wong and Brown, 2007). The qualitative analysis of search strategies also indicated that aged WT animals were equally able to acquire spatially precise search strategies compared to adults, further supporting the notion that the minimally enriched environment is promoting healthy cognitive aging. In contrast, even under these favorable conditions, aged β2-/- mice did not acquire the use of place-specific and allocentric strategies, revealing significant, but a subtler, deficit in spatial learning that is not detectable in the conventional measures of latency to locate the platform, or probe test.

This deficit was also evident in the more demanding assessment of spatial cognition, the reverse water maze task, where the hidden platform is moved after the first acquisition phase, whereas all extramaze cues remain unchanged. This task evaluates the ability to cope with changes in behaviorally relevant stimuli of the environment, and requires a balance between establishing stable cognitive schemata while maintaining the capacity to flexibly alter these in the face of new information (Walsh et al., 2011; Dong et al., 2013). In this more challenging task, old β2-/- mice performed significantly worse than the other three groups reflecting a deficit in extinction of the previously learned platform position (Zanardi et al., 2007). Importantly, in this task the reference memory for the new location was deficient already in *adult* β2-/- animals, hence providing an early behavioral indication of an abnormal aging process (Marubio and Paylor, 2004). Given that reversal learning and memory are considered as measures of executive function that depend on the integrity of the prefrontal cortex (Packard and Knowlton, 2002) these data are in line with morphological studies showing that pyramidal cell microanatomy in these regions is compromized already in adulthood (Ballesteros-Yáñez et al., 2010; Konsolaki and Skaliora, 2015b) and deteriorates further with aging (Konsolaki and Skaliora, 2015b).

Taken together, these results confirm that the lack of high affinity nicotinic receptors leads to age-related deficits in spatial cognition, even under the minimally enriched housing conditions employed in the present study. The inability of old β2-/- mice to acquire global strategies during the acquisition task may also explain their deficient performance in reversal learning. These deficits are consistent with these animals’ difficulty to deal with conflicting motivations (Serreau et al., 2011) and their inability to modify their routine behavior in the face of changed conditions (Granon et al., 2003).

#### Recognition Memory

In order for an animal to be a valid model for accelerated/premature cognitive aging the decline should not be confined to the realm of spatial learning, but should generalize across different cognitive capacities. To assess whether this applies to β2-/- mice we used the NORT, a behavioral assay of memory that engages the cholinergic system (Sambeth et al., 2007; Savage et al., 2011; Cai et al., 2012; Okada et al., 2015), and relies primarily on a rodent’s innate exploratory behavior and not on externally applied rules or reinforcement (as in the water maze task). Such tests evaluate the ability to recognize a previously presented stimulus and are thought to constitute the core of animals’ models of human amnesias (Gaffan, 1974; Zola-Morgan et al., 1982; Baxter, 2010). In agreement to the results of the MWM task, adult β2-/- mice manifested normal recognition and retention of the familiar object, indicating that loss of high-affinity nicotine receptors *per se* is not sufficient to cause learning deficits. In contrast, aged mutant animals were not able to discriminate the new from the familiar object, lending further support to the hypothesis that the chronic absence of β2-nAChRs is associated with pathological cognitive aging.

It is also noteworthy that, in this task as well, the performance of old WT animals failed to show the expected age-related decline (Baxter, 2010; Burke et al., 2010), further supporting the notion that the minimally enriched environment employed in our study promotes successful cognitive aging.

Taken together our data indicate that mutant mice lacking high affinity nicotinic receptors do not exhibit explicit cognitive decline as adults but instead become cognitively impaired when they age, suggesting that β2-nAChRs contribute to the maintenance of cognitive performance during aging.

### β2-/- Mice Display Changes in Spontaneous Behaviors Before the Onset of Cognitive Deficits

Having documented the pathological cognitive decline of β2-/- mice, we aimed to identify early behavioral signs that could presage an accentuated aging process. Studies in humans have shown that alterations in behavior and ADL can precede cognitive decline by many months or years (Dias et al., 2015) and thus could be useful for identifying people at risk of developing dementia (Njegovan et al., 2001). However, to our knowledge, such studies have not been performed in animals. Assessment of ADL in rodents has been modeled in several species-specific behavioral tasks, such as hoarding, marble burying, burrowing and nesting (Deacon, 2012; Deacon et al., 2012). These are all innate behaviors with diverse functions, such as securing food supply for times of emergency (hoarding; Deacon et al., 2007); hiding small pellets as a measure of repetitive behavior (marble burying; Thomas et al., 2009); displacement of food from a tube (burrowing; Deacon, 2006c) and constructing a shelter for thermoregulation and safety (nesting; Deacon, 2006b). These behaviors are performed by both male and female animals and are not affected by laboratory conditions (Deacon et al., 2012). Here, we examined all four tasks and our data reveal varying effects of age and genotype, suggesting distinct regulatory mechanisms.

In the first two tasks (hoarding and marble burying) mutant animals behaved indistinguishably from the WT group. In contrast, β2-/- mice exhibited significant deficits in the latter two tasks (burrowing and nesting). The decline in burrowing performance was only evident in aged animals, consistent with a pathological aging process. This result cannot be attributed to purely motor deficits as old mice of both genotypes displayed similar locomotor activity. Given that burrowing is dependent on an intact hippocampus (Deacon et al., 2002), the age-dependent loss of hippocampal pyramidal neurons found in old β2-/- mice (Zoli et al., 1999) is likely to give rise to the observed decrease in burrowing behavior. In contrast to the age-dependent decline in burrowing, the deficit in nest construction was present already in adult animals and could reflect an early indication of a pathological aging process.

The final assessment of the animals’ spontaneous behaviors was their locomotion and exploratory activity in a novel environment. When placed in unfamiliar surroundings, mice will initially cover large distances as they explore the environment and then gradually reduce their activity levels as they habituate. This habituation process is defined as “a response decrement with repeated or continuous presentation of indifferent stimuli, which is not dependent on muscle fatigue or receptor adaptation” (Thompson and Spencer, 1966); and represents one of the most elementary forms of learning, in which the decreasing exploration, as a function of time and/or repeated exposure to the same environment, is taken as an index of memory (Thiel et al., 1998; Pedrazza et al., 2007). Open field exploration and habituation are related to the hippocampus and its cholinergic input (Day et al., 2001) and deficits in these processes could reflect compromised brain networks with heightened vulnerability to the process of aging.

Our results clearly demonstrate an impaired habituation in β2-/- mice, which had not been previously recognized/appreciated. Earlier studies on these animals had reported a hyperactive phenotype on the basis of increased locomotion over 30 min periods (Avale et al., 2008; Bourgeois et al., 2012). Our data confirm such an increase in total distance covered, but demonstrate that this increase does not manifest in the initial stages of the open field task but is confined only to the later 30 min period, revealing a profound deficit to habituate to a novel environment (Konsolaki and Skaliora, 2015a). Consistent with this interpretation, when locomotion was examined for short time periods β2-/- mice covered the same distances as WT controls (Guillem et al., 2011).

Previous studies have shown that different locomotion behaviors such as navigation (fast movements) and exploration (slow movements) reflect different ways of information gathering (Granon et al., 2003; Maskos et al., 2005; Granon and Changeux, 2006; Avale et al., 2008; Wiklund et al., 2008). Exploratory activity is a spontaneous behavior that does not involve any explicit reinforcement and serves to gather and store spatial information which is required for flexible navigational processes (Granon et al., 2003). Hence, a detailed assessment of the animals’ behavior in a novel environment could provide important insights on a basic cognitive structure, which can be used to phenotype animals at risk of cognitive decline. Our data reveal that exploration is compromized in β2-/- mice already in adulthood, and throughout the entire duration of the open field task. This suggests that these animals fail to exhibit the normal reduction in overall mobility, not because they have higher levels of activity, but because they are unable to perform a comprehensive or adequate investigation of the surroundings, and hence to efficiently gather and to store spatial information. Such a deficit is likely to affect other cognitive processes and could be considered a poor prognosis for successful cognitive aging.

## Conclusion

The results of the present study indicate that high affinity nicotinic receptors are not essential for the animals’ learning ability or memory performance, in either spatial or non-spatial tasks. However, the lack of these receptors is associated with significant age-dependent cognitive decline in old animals, suggesting a link between a deficient nicotinic system and pathological cognitive aging. In addition, β2-/- animals exhibit alterations in exploratory behavior and habituation to a novel environment, as well as nest construction already in adulthood, suggesting these spontaneous activities could provide early indications of an accelerated aging process, that could be useful in screening animals at risk for developing dementia.

## Author Contributions

EK designed and performed research, analyzed the data and wrote the paper, PT contributed analytic tools, AP contributed to the set up of the behavioral facility and the design of pilot experiments, AS provided input for the behavioral protocols and interpretation of the data, IS conceived and designed research and wrote the paper.

## Conflict of Interest Statement

The authors declare that the research was conducted in the absence of any commercial or financial relationships that could be construed as a potential conflict of interest.

## Supplementary Material

**FIGURE S1 | Example of the velocities histogram.** Gray bars indicate the occurrence of velocities at 1 cm/sec bins, black thin lines are the Gaussian distributions fitted to the data and the red thick line corresponds to the mixture density function. The blue vertical lines indicate the Low- and High Threshold values that separate the range of velocities into three types of movement (Exploration = slow, local movements; Navigation = fast, large movements; and Intermediate).
