# Supplementary figures and images for: Early Signs of Pathological Cognitive Aging in Mice Lacking High-Affinity Nicotinic Receptors
Source: Front Aging Neurosci. 2016 Apr 27;8:91. doi: 10.3389/fnagi.2016.00091 (PMC4846665; doi:10.3389/fnagi.2016.00091)

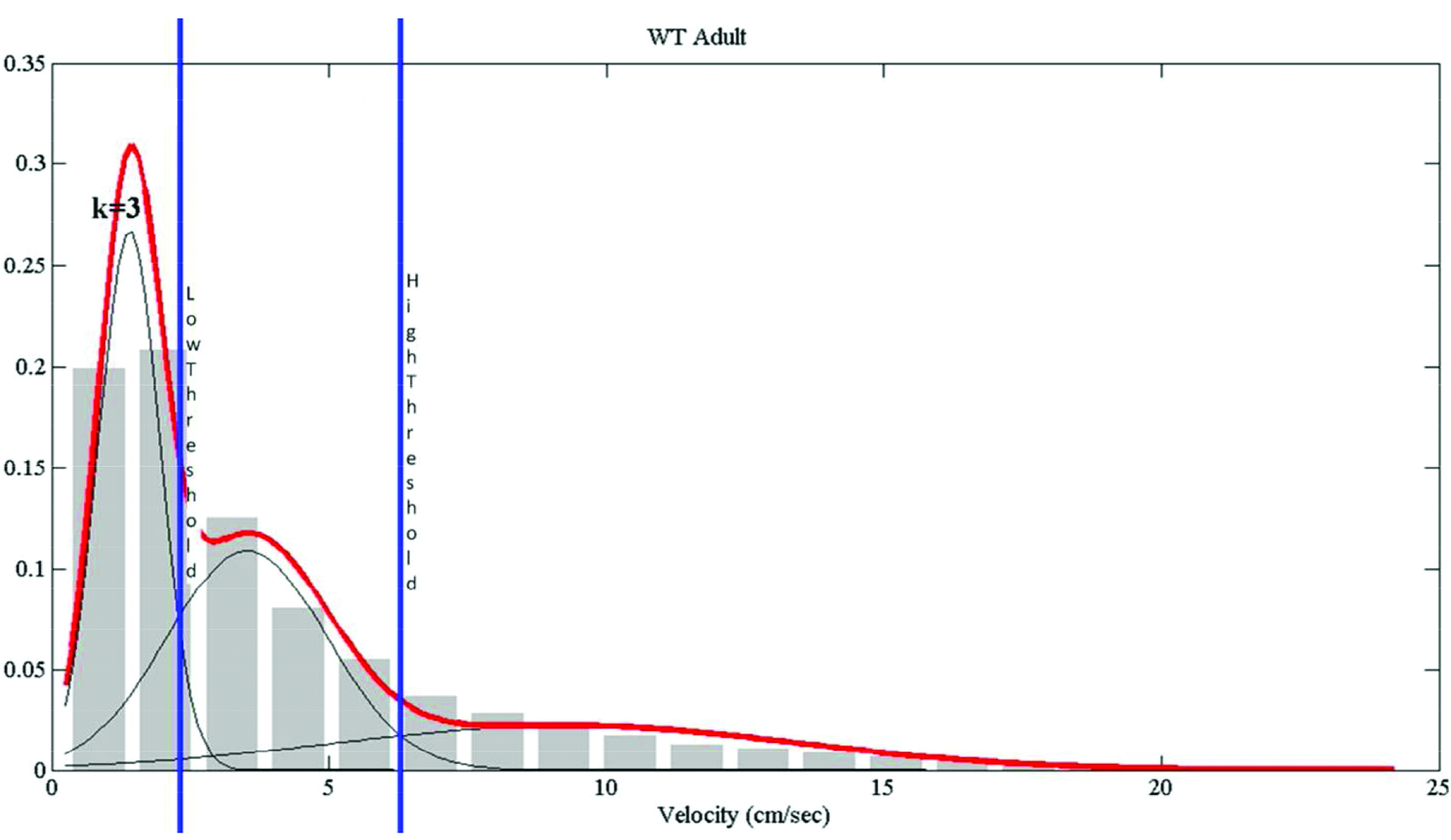

Supplement: Supplementary file 1 [file Image_1.TIF]
