# Supplementary material for: Early Signs of Pathological Cognitive Aging in Mice Lacking High-Affinity Nicotinic Receptors
Source: Front Aging Neurosci. 2016 Apr 27;8:91. doi: 10.3389/fnagi.2016.00091 (PMC4846665; doi:10.3389/fnagi.2016.00091)
Supplement: Supplementary file 2 [file Presentation_1.PDF]

Open field exploration and navigation measurements

In order to estimate the time spent in fast and slow movements in an un-biased way, we used the following protocol:

1. Calculation of the instantaneous velocity (cm/sec) for each video file and creation of a database that included the velocity time-series for every animal at the same age&genotype group.

2. Generation of MatLab code in order to classify velocity values for each animal into three categories: slow movements or 'exploration', fast movements or 'navigation' and 'intermediate'. Exploration was defined as movement with velocities up to a specific velocity value (from hereafter called LowThreshold). The 'intermediate' group was defined as movement with velocities between the aforementioned LowThreshold value and a second (higher) velocity value (called from now on HighThreshold). Navigation was defined as movement with velocities from the aforementioned HighThreshold and above. The two threshold leves (Low and HighThreshold) were estimated as follows:

   2.1. For each animal of a specific age and genotype, we calculated a histogram of velocity values, where we first performed a histogram Bandwidth Optimization in order to determine the optimal number and size of bins (this was done in order to avoid generalization biases, as different animals could have different velocity ranges). This step provided us with a velocity density estimation which was used for the calculation of the Low and HighThresholds and the classification of velocities.

   2.2. Taking into account the three classes of movement, we fit to each histogram a Mixture of Gaussians Model, which consisted of three Gaussian distributions. The model was fitted to the data via the Expectation-Maximization (EM) algorithm.

   2.3. After determining the properties of each Gaussian distribution (i.e. their corresponding mean, covariance and mixture proportion) we calculated the Bayes discriminant value between the three classes. This provided the two values corresponding to the Low and HighThreshold for each animal.

2.4. To determine the final threshold values for each group we calculated the average values among all animals of the same age and same genotype.

3. Once the Low and HighThreshold values were determined, we calculated the percentage of time in each trial the animals spent in each type of movement.

4. To compare between experimental groups we used the threshold values of the wildtype adult group as baseline, in order to enable the simultaneous testing for age and genotype effects.
